# Development and Clinical Evaluation of a Web-Based Upper Limb Home Rehabilitation System Using a Smartwatch and Machine Learning Model for Chronic Stroke Survivors: Prospective Comparative Study

**DOI:** 10.2196/17216

**Published:** 2020-07-09

**Authors:** Sang Hoon Chae, Yushin Kim, Kyoung-Soub Lee, Hyung-Soon Park

**Affiliations:** 1 Graduate School of Medical Science and Engineering Korea Advanced Institute of Science and Technology Daejeon Republic of Korea; 2 Major of Sports Health Rehabilitation Cheongju University Cheongju Republic of Korea; 3 Department of Mechanical Engineering Korea Advanced Institute of Science and Technology Daejeon Republic of Korea

**Keywords:** home-based rehabilitation, artificial intelligence, machine learning, wearable device, smartwatch, chronic stroke

## Abstract

**Background:**

Recent advancements in wearable sensor technology have shown the feasibility of remote physical therapy at home. In particular, the current COVID-19 pandemic has revealed the need and opportunity of internet-based wearable technology in future health care systems. Previous research has shown the feasibility of human activity recognition technologies for monitoring rehabilitation activities in home environments; however, few comprehensive studies ranging from development to clinical evaluation exist.

**Objective:**

This study aimed to (1) develop a home-based rehabilitation (HBR) system that can recognize and record the type and frequency of rehabilitation exercises conducted by the user using a smartwatch and smartphone app equipped with a machine learning (ML) algorithm and (2) evaluate the efficacy of the home-based rehabilitation system through a prospective comparative study with chronic stroke survivors.

**Methods:**

The HBR system involves an off-the-shelf smartwatch, a smartphone, and custom-developed apps. A convolutional neural network was used to train the ML algorithm for detecting home exercises. To determine the most accurate way for detecting the type of home exercise, we compared accuracy results with the data sets of personal or total data and accelerometer, gyroscope, or accelerometer combined with gyroscope data. From March 2018 to February 2019, we conducted a clinical study with two groups of stroke survivors. In total, 17 and 6 participants were enrolled for statistical analysis in the HBR group and control group, respectively. To measure clinical outcomes, we performed the Wolf Motor Function Test (WMFT), Fugl-Meyer Assessment of Upper Extremity, grip power test, Beck Depression Inventory, and range of motion (ROM) assessment of the shoulder joint at 0, 6, and 12 months, and at a follow-up assessment 6 weeks after retrieving the HBR system.

**Results:**

The ML model created with personal data involving accelerometer combined with gyroscope data (5590/5601, 99.80%) was the most accurate compared with accelerometer (5496/5601, 98.13%) or gyroscope data (5381/5601, 96.07%). In the comparative study, the drop-out rates in the control and HBR groups were 40% (4/10) and 22% (5/22) at 12 weeks and 100% (10/10) and 45% (10/22) at 18 weeks, respectively. The HBR group (n=17) showed a significant improvement in the mean WMFT score (*P*=.02) and ROM of flexion (*P*=.004) and internal rotation (*P*=.001). The control group (n=6) showed a significant change only in shoulder internal rotation (*P*=.03).

**Conclusions:**

This study found that a home care system using a commercial smartwatch and ML model can facilitate participation in home training and improve the functional score of the WMFT and shoulder ROM of flexion and internal rotation in the treatment of patients with chronic stroke. This strategy can possibly be a cost-effective tool for the home care treatment of stroke survivors in the future.

**Trial Registration:**

Clinical Research Information Service KCT0004818; https://tinyurl.com/y92w978t

## Introduction

Stroke is a major cause of disability in adults. About 13.7 million cases of stroke occur each year globally, but half of the patients are unable to restore enough upper extremity function required for daily living [1,2]. The rehabilitation required after stroke has been limited to the first 3 to 6 months of hospitalization following the stroke [3]. For the best recovery following stroke and prevention of recurrence, stroke survivors need ongoing home rehabilitation [4-7]. Previous literature has proven that continued home rehabilitation can activate neuroplasticity in chronic poststroke patients and result in greatly enhanced clinical outcomes [8-10]. In addition, the need for a high-quality home health care system is drawing greater attention with the recent COVID-19 pandemic. The major barriers in delivering high-quality home rehabilitation services are high cost and labor intensiveness [11,12]. Therefore, socioeconomically deprived people are less likely to receive high-quality rehabilitation care and more likely to experience recurrence and poor quality of life [13,14]. The burdensome labor of home care also puts the care giver and receiver at risk for poor mental health and depression [15,16].

To overcome the barriers for home rehabilitation, potential technology-enabled solutions have been suggested. For example, there are two kinds of technology used as solutions (vision-based solution and wearable sensor–based solution). The vision-based approach (eg, interactive television or Kinect) could be easier to use since it does not require any wearing of devices [17-21]. A vision-based system can only be used within a limited range of space, whereas wearable systems can be used anywhere, which would be advantageous for promoting the frequency of use [22,23].

To promote the frequency of use, we developed an upper limb home-based rehabilitation (HBR) system using wearable sensors embedded in a commercial smartwatch. A machine learning (ML) algorithm implemented by a convolutional neural network (CNN) was used to recognize four kinds of home exercise activities. While participants perform these home exercises, the HBR system makes it possible to share their home exercise data with therapists at remote locations. It helps therapists to encourage and communicate with chronic stroke survivors.

We conducted a prospective comparative study to evaluate the effectiveness of our HBR system. As the long-term goal of this study, we intended to investigate the benefits of using artificial intelligence–based HBR compared with those of conventional therapy. Herein, we compared the clinical outcomes of an experimental (HBR) group using the HBR system with those of a control group performing conventional home exercises. We hypothesized that the HBR group would show enhanced clinical outcomes compared with the control group [24]. This paper elaborates on the technological advancements pertaining to the detection of home exercise activities using a smartwatch (the ML model) and the results from a clinical trial.

## Methods

### Development of an HBR System

#### Overview of an HBR System

**Figure 1:**
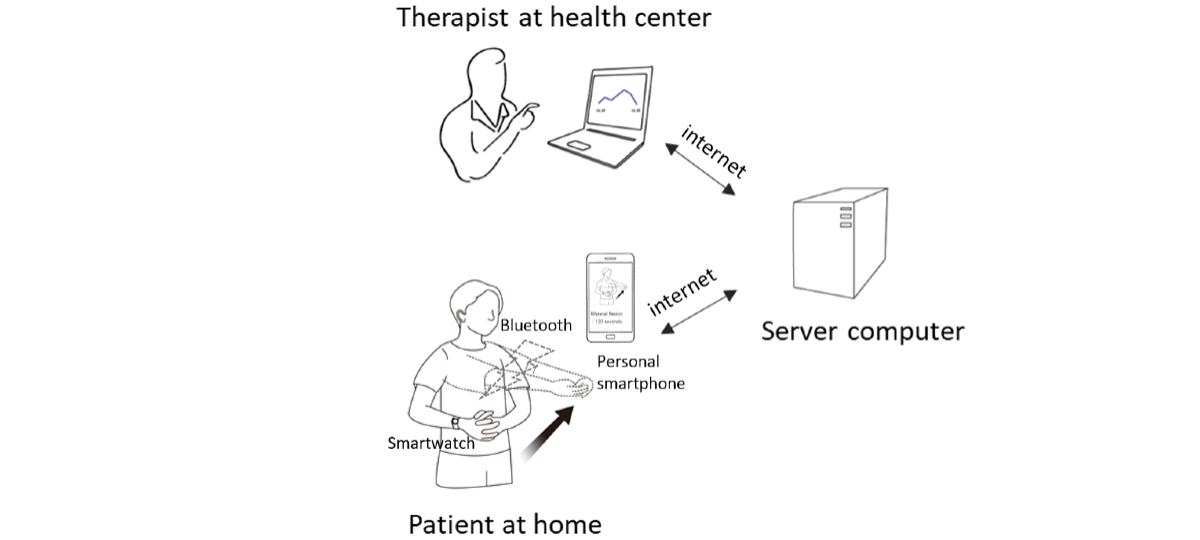
Overview of the home-based rehabilitation system.

We implemented an HBR system that can connect patients and therapists at a distance. Figure 1 presents an overview of our HBR system. To make the interface simple and user-friendly, we used a commercial smartwatch (watch style W270, LG,) that can be connected to a personal smartphone after installing a custom-programmed app. In our system, the smartwatch, which includes an inertial measurement unit (IMU) sensor, sent sensor data to the smartphone via Bluetooth communication while patients were doing exercise. The personal smartphone served as a platform for receiving sensor data, classifying the data, and transmitting the results to a server computer via the internet (Multimedia Appendix 1). The apps for the smartwatch and smartphone were developed using the Android Software Development Kit (Android Studio 2.3, Google).

#### Selection of Home Rehabilitation Exercise Tasks

**Figure 2:**
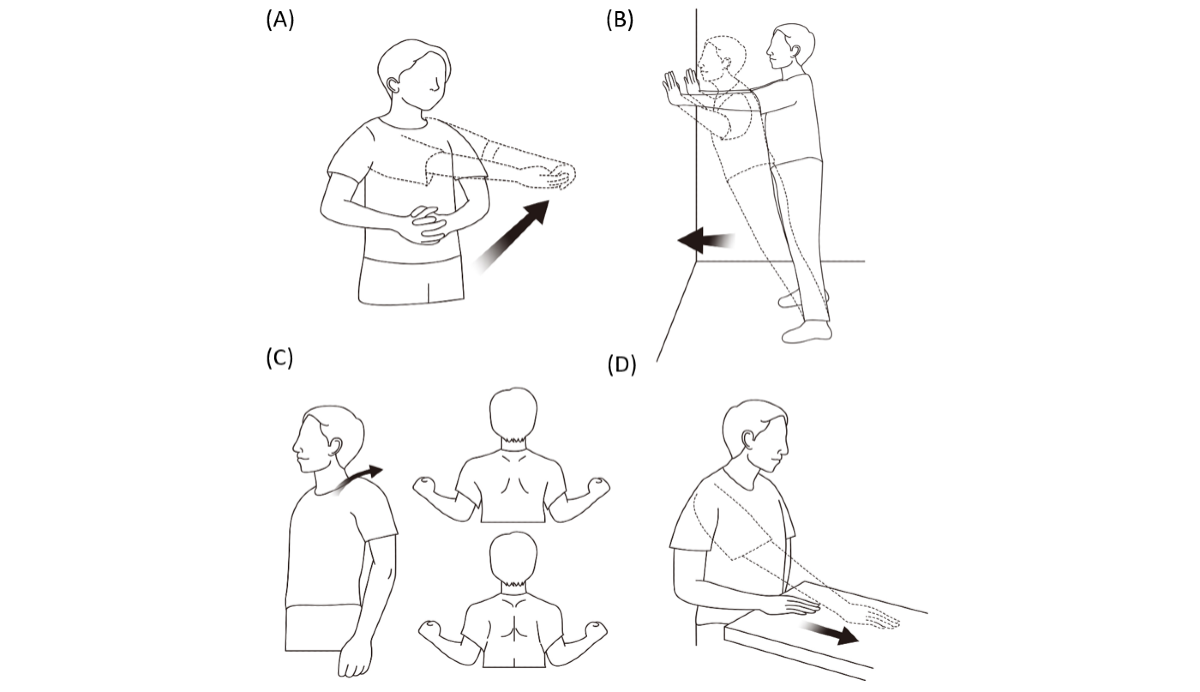
Home rehabilitation exercises for the upper limbs. (A) Bilateral shoulder flexion; (B) Wall push exercise; (C) Active scapular exercise; (D) Towel slide exercise.

We selected four exercise tasks based on bilateral movement therapy, which is called bilateral arm training rehabilitation. Previous literature has shown that bilateral arm training can induce reorganization in contralateral motor networks by interhemispheric crosstalk and evoke functional recovery of the upper extremities in chronic stroke survivors [25,26]. As shown in Figure 2, the following exercises were selected: (1) bilateral shoulder flexion with both hands interlocked; (2) wall push exercise; (3) active scapular exercise; and (4) towel slide exercise.

#### Machine Learning Algorithm for Home Exercise Recognition

There are various kinds of deep learning algorithms for human activity recognition [27,28]. Among them, we selected the CNN as it has been reported to be highly accurate in human activity recognition and simpler than other algorithms since it does not need feature extraction [29,30]. We made a program for building the ML model with Python script (Python 3.5, Python Software) and CNNs in the TensorFlow platform (Tensorflow 1.7.0, Google).

**Figure 3:**
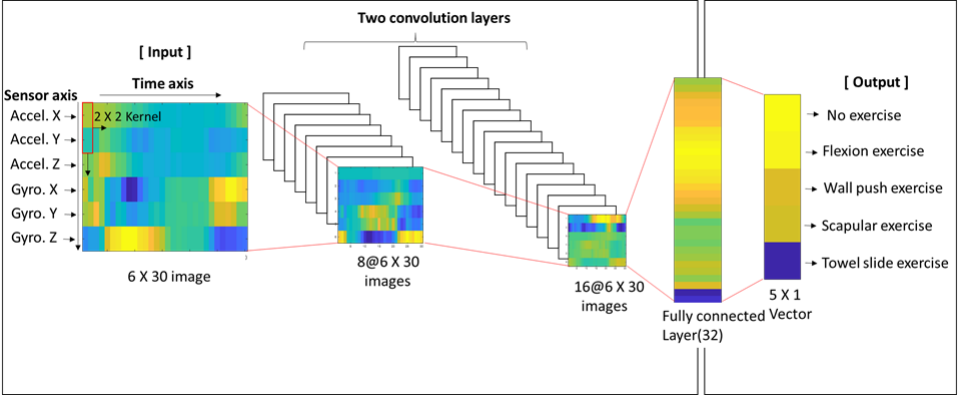
Baseline convolutional neural network architecture.

The kinematic data structure from the IMU sensor consists of three-axis (x, y, and z) accelerometer and gyroscope data. When a patient is exercising while wearing the smartwatch, the accelerometer and gyroscope of the IMU sensor measure acceleration and velocity during the exercise. Since all sensor data are in a time series sampled at 10 Hz, the entire data can be represented by a two-dimensional matrix with a time axis (horizontal) and a sensor axis (vertical) as shown in Figure 3. We used the sliding time window method and applied a 3-second time window according to the experimental results that compared the performance of the ML algorithm at various time windows [31].

The training data essential for implementing the ML algorithm were obtained on the first day of the meeting. As it was difficult to meet patients with chronic stroke due to the difficulty of moving, we gathered the data on the same day just after explaining about the four kinds of home exercises. Participants were asked to repeat them 15 times in two sessions wearing a smartwatch.

Figure 3 reflects our baseline CNN architecture. Two convolution layers, which have 8 and 16 feature maps, are followed by a fully connected layer that has 32 nodes. Rectified units are employed as activation functions, and SoftMax functions are used for evaluating the final five output node values.

We experimented with the following two types of ML models: a ML model built with the personal data set and another ML model built with the total data set. The personal data set was composed of the exercise data of the user, whereas the total data set consisted of all participants’ exercise data including the user. In order to evaluate the accuracy of each model, we applied a cross-validation test.

#### Cross-Validation Test for Accuracy Comparison

A five-fold cross-validation test was performed to test the accuracy of the ML model in recognizing exercise tasks. We divided the data into one test data set and four training data sets. The training data sets were used to build the ML model, and the test data set was used to determine the accuracy of the ML model built. Thus, we compared the accuracy of the model created by personal data versus total data. Additionally, we compared the accuracy between models based on each sensor data (accelerometer only, gyroscope only, and accelerometer and gyroscope combined) to determine which sensor data are most accurate for exercise prediction. Accuracy was calculated by using the following formula:

Accuracy = (TP + TN) / (TP + TN + FP + FN)

where TP is true positive, TN is true negative, FP is false positive, and FN is false negative.

#### Development of Mobile Apps

We implemented the following three different android apps: (1) smartwatch app; (2) smartphone app for patients; and (3) smartphone app for physiotherapists (Android Studio 2.3, Google). The smartwatch app is designed to transmit sensor data to a smartphone as soon as the exercise button on the smartphone is pressed and to stop transmission when the use of the app on the smartphone has ended. There is no start or stop button on the smartwatch. We made the smartphone app automatically close as soon as the android app of the smartphone shuts down. The smartphone app for patients acts as a platform for starting the smartwatch, detecting home rehabilitation, and transmitting exercise time data to the server computer. The personalized ML model embedded in the smartphone app recognizes the type of exercise that the participant is doing. After recognition of the exercise, the smartphone calculates and transmits the exercise time via the internet. It also shows the personal rehabilitation time of the previous 3 days on pressing a button in the app. Lastly, the smartphone app for physiotherapists provides the physiotherapists with the rehabilitation status of all enrolled patients for the past 1 month for convenient statistical evaluation.

### Clinical Trial: Prospective Comparative Study

#### Experiment Design

We performed a clinical trial in two local health care centers located in two cities in South Korea (Cheongju [control group] and Daejeon [HBR group]). In the two centers, we recruited 12 and 26 patients with chronic stroke, respectively. The inclusion criteria were as follows: (1) age 40 to 70 years, (2) mild to moderate neurologic deficit with hemiplegia, (3) more than 6 months after the onset of stroke, (4) 24 points or more in the Korean version of the Mini-Mental State Examination (K-MMSE), and (5) ability to understand the procedures and communicate with the supervisor. The exclusion criteria were as follows: (1) arthritis of the glenohumeral joint, (2) rotator cuff tear, (3) cervical root syndrome, (4) subluxation of the shoulder joint, (5) reluctance to follow the home exercise regimens of this study, and (6) no smartphone with Android OS.

**Figure 4:**
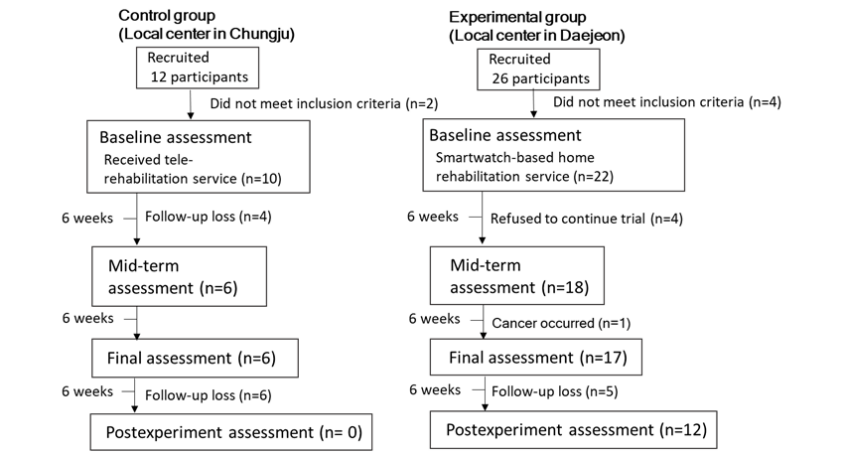
Time flow of the study.

Figure 4 shows the time flow of the study. According to the criteria, we excluded two patients in the control group owing to shoulder pain during exercise. In the HBR group, four patients were excluded (one patient had rotator cuff repair surgery previously, one had shoulder subluxation, and two had shoulder pain during exercise). After excluding those patients, 10 and 22 patients were initially enrolled in the control and HBR groups, respectively. While performing the home rehabilitation program, four patients in the control group dropped out. Drop-out was determined by a therapist who undertook the task of home-based rehabilitation for participants who did not respond to phone calls. In the HBR group, four patients gave up using our HBR system, as they were unfamiliar with the information technology devices and experienced difficulties in their usage. One patient missed the rehabilitation owing to deterioration caused by other underlying diseases. Finally, six patients in the control group and 17 patients in the HBR group completed the protocol at 12 weeks. To determine the changes in clinical scores, we tried to conduct an assessment at 6 weeks after the final assessment (18 weeks). Participants in the control group did not respond to our call, whereas participants in the HBR group cooperated with the requirement for assessment.

All patients in the control group received personal education about the four exercise tasks for 30 minutes at the beginning of study enrollment. In the control group, the participants received a printed handout to remind them about how to perform the four exercise tasks. In contrast, participants in the HBR group received the same education and were given a smartwatch, and the HBR apps were installed on their own smartphones on the first day of the meeting. During the education, we acquired learning data for the ML algorithm in the HBR group. The physiotherapist taught each individual patient how to perform the four exercise tasks, and the training data were labeled manually while the patient was practicing each of the four tasks. In other words, with the smartwatch worn, participants were asked to repeat each home exercise 15 times in two sessions. In total, data were collected for 120 exercise attempts.

In both groups, weekly calls were made by the same therapists [17]. To avoid bias from the examiner, two therapists equally divided the participants present in both groups and managed them. They encouraged the participants to perform home exercise and answered any questions regarding how to perform the home exercise from participants in both groups. Since the control group did not use any sensor at home, one additional question was asked regarding how much time the control participants spent on home exercise.

In the HBR group, the participants were able to obtain their own home exercise results, and it was possible for the therapist to access the data of all the participants. Thereafter, the physiotherapist communicated with the participants and encouraged them based on the home exercise data collected by the HBR system (Multimedia Appendix 2).

All participants were asked to come to the local health care centers for outcome evaluation, and two physical therapists at each center conducted the clinical assessments at 0 weeks (baseline), 6 weeks (mid-term), and 12 weeks (final). In addition, we conducted one more assessment at 18 weeks, which was a 6-week follow-up after the completion of the home rehabilitation program, to examine the change after our home rehabilitation program. For functional scoring, the Fugl-Meyer Assessment of Upper Extremity (FMA-UE) and Wolf Motor Function Test (WMFT) were used. We also evaluated psychologic depression using Beck Depression Inventory (BDI), grip power using a dynamometer (Patterson Medical), and shoulder range of motion (ROM) angle using a goniometer.

#### Statistical Analysis

We used descriptive statistics to characterize the demographics and analyzed the difference between the control and HBR groups at baseline using the Mann-Whitney *U* test. We compared the clinical results of functional recovery (WMFT and FMA-UE), grip power, BDI, and ROM using the Friedman test. As a post-hoc analysis, the Wilcoxon signed-rank test with Bonferroni correction was used. SPSS software was utilized for all statistical analyses (SPSS statistics 25, IBM Corp).

The sample size was determined according to a previous study (using virtual reality home training) related to this study [32]. Based on the result of a mean ROM increase of 41.67° (SD 22.29°), we calculated the sample size using G*Power software (two-tailed; α error, .05; power [1–β error], 0.8; effect size, 1.869; loss rate, 10%). This power analysis showed a sample size of 6.

## Results

### Accuracy Results of the HBR System

It was impossible to detect the accuracy of the home exercise activity logs as the ground truth of home exercise motion of the participants was unknown owing to matters pertaining to personal privacy. Instead, we attempted to ascertain what types of sensor data can detect home exercise activities most accurately via a cross-validation test.

The results shown in Table 1 represent the accurate values that were calculated by the cross-validation test with different ML models depending on various input data and sensor data. With regard to the input data, the ML model trained by accelerometer combined with gyroscope data had the best accuracy compared with other models. In particular, the ML model developed using personal data (99.9%) was more accurate than the model developed using total data (95.8%), although the amount of personal data was much smaller than that of total data.

Multimedia Appendix 3 shows the results of the cross-validation test.

**Table 1:** Accuracy of the convolutional neural network model according to exercise.

Exercise	Personal data (%)			Total data (%)		
	A^a^	G^b^	A+G^c^	A	G	A+G
No exercise	100 (1224/1224)^d^	95.8 (1172/1224)	100 (1224/1224)	97.4 (1192/1224)	97.8 (1197/1224)	100 (1224/1224)
Bilateral flexion	98.5 (1103/1120)	97.8 (1095/1120)	99.0 (1109/1120)	96.1 (1076/1120)	98.1 (1099/1120)	97.2 (1089/1120)
Wall push	99.0 (1014/1024)	93.7 (959/1024)	100 (1024/1024)	93.8 (960/1024)	86.5 (886/1024)	93.2 (954/1024)
Active scapula	93.0 (1030/1108)	97.6 (1081/1108)	100 (1108/1108)	92.2 (1022/1108)	87.0 (964/1108)	93.0 (1030/1108)
Towel slide	100 (1125/1125)	95.5 (1074/1125)	100 (1125/1125)	94.2 (1060/1125)	88.7 (998/1125)	95.5 (1074/1125)
Total	98.1 (5496/5601)	96.0 (5381/5601)	99.9 (5590/5601)	94.8 (5310/5601)	91.8 (5144/5601)	95.8 (5371/5601)

^a^Accelerometer data.

^b^Gyroscope data.

^c^Accelerometer combined with gyroscope data.

^d^Indicates the value of correct samples divided by total samples.

### Results of the Clinical Trial

The study was approved by the Institutional Review Board (IRB no.: IRB-17-299). Informed consent was obtained from all participants. This study was supported by the Korea Advanced Institute of Science and Technology-funded Global Singularity Research Program. Patients were recruited from March 2018 to September 2018, and home exercise data were collected until February 2019. As of March 2019, we enrolled 23 stroke survivors for data analysis. Drop-out rates in the control and HBR groups were 40% (4/10) and 22% (5/22) at 12 weeks and 100% (10/10) and 45% (10/22) at 18 weeks, respectively.

Table 2 presents the demographics and baseline assessment findings. There were no relevant differences between the two groups.

To evaluate exercise compliance at home, in the control group, a telephone survey was the only approach to determine the home exercise activities of participants. Thus, we called them and asked how much time they exercised at home and encouraged exercise. We found that they performed home exercise for about 13.6 (SD 4.85) min/day. However, the numbers obtained for the control group might not be accurate owing to the limitations of a verbal survey. In contrast, in the HBR group, the home exercise results of all the participants were provided by the smartphone app. Thus, we encouraged participants to perform home exercise based on the data.

**Table 2:** Patient demographics and baseline assessment in the control and home-based rehabilitation groups.

Characteristic		Control group (n=6), mean (SD)	HBR^a^ group (n=17), mean (SD)	*P* value^b^
Age (years)		64.5 (9.6)	58.3 (9.3)	.25
**Functional assessment test**				
	WMFT^c^	38.8 (25.6)	39.7 (22.2)	.91
	FMA-UE^d^	29.0 (14.2)	36.6 (18.6)	.35
	Grip power (kg)	11.7 (11.6)	13.3 (12.7)	.75
BDI^e^		24.2 (11.2)	17.88 (14.7)	.28
**Shoulder ROM^f^**				
	Flexion	82.0 (59.07)	74.5 (45.3)	.91
	Extension	40.8 (19.6)	28.7 (21.0)	.11
	Internal rotation	50.8 (31.2)	50.43 (24.5)	.51
	External rotation	23.4 (28.1)	16.84 (17.69)	.97

^a^HBR: home-based rehabilitation.

^b^*P* values were calculated with the Mann-Whitney *U* test.

^c^WMFT: Wolf Motor Function Test.

^d^FMA-UE: Fugl-Meyer Assessment of Upper Extremity.

^e^BDI: Beck Depression Inventory.

^f^ROM: range of motion.

On average, participants in the HBR group performed bilateral flexion exercise for 7.27 (SD 10.1) min/day, wall push exercise for 3.76 (SD 9.01) min/day, active scapula exercise for 4.82 (SD 9.62) min/day, and towel slide for 6.70 (SD 11.87) min/day. In total, home exercise was performed for an average of 22.57 (SD 37.69) min/day.

Table 3 presents the clinical results at the baseline, mid-term (6 weeks), and final assessments (12 weeks). In total, 23 individuals with chronic stroke completed this research (control: 6; HBR: 17). In the HBR group, the WMFT, BDI, and shoulder ROM of flexion and internal rotation showed relevant progression (Multimedia Appendix 4). However, FMA-UE showed no significant difference (*P*=.46). In the control group, there was no significant difference, except for the ROM of internal rotation (*P*=.03). In both groups, there was no relevant difference in the grip power test.

We tried to determine the change in clinical results after the completion of the home rehabilitation program, which had a duration of 12 weeks. Thus, we compared the clinical outcomes at the final assessment (12 weeks) with that at 6 weeks after removing the HBR system (18 weeks). However, there was no relevant difference (Multimedia Appendix 5).

**Table 3:** Clinical results in the control and home-based rehabilitation groups during the experiment.

Characteristic		Control group (n=6)				HBR^a^ group (n=17)			
		0 weeks, mean (SD)	6 weeks, mean (SD)	12 weeks, mean (SD)	*P* value^b^	0 weeks, mean (SD)	6 weeks, mean (SD)	12 weeks, mean (SD)	*P* value^b^
**Functional assessment test**									
	WMFT^c^	38.8 (25.6)	40.3 (25.7)	42.2 (22.8)	.69	39.7 (22.7)	40.5 (23.6)	42.5 (23.7)	.02
	FMA-UE^d^	29.0 (14.2)	30.0 (14.2)	28.5 (16.1	.72	36.6 (18.7)	37.5 (18.4)	38.5 (18.3)	.46
	Grip power (kg)	11.7 (11.6)	11.0 (10.4)	10.9 (10.3)	.47	13.3 (12.7)	12.9 (12.0)	14.8 (12.1)	.34
BDI^e^		24.2 (11.2)	10.0 (8.6)	8.8 (7.2)	.11	17.9 (14.7)	10.0 (8.8)	8.0 (9.9)	.06
**Shoulder ROM^f^**									
	Flexion	82.0 (59.1)	90.6 (65.3)	87.5 (61.0)	.21	74.5 (45.3)	93.9 (52.3)	94.7 (48.9)	<.001
	Extension	40.8 (19.6)	29.5 (18.9)	32.8 (20.4)	.38	28.7 (21.0)	31.5 (16.2)	34.7 (19.9)	.16
	Internal rotation	50.8 (31.2)	48.5 (28.6)	57.3 (32.0)	.03	50.4 (24.5)	70.3 (28.3)	63.5 (26.9)	.001
	External rotation	23.4 (28.1)	23.6 (30.0)	26.6 (27.7)	.76	16.8 (17.7)	15.4 (18.1)	16.9 (18.4)	.20

^a^HBR: home-based rehabilitation.

^b^Overall *P* values were calculated with the Friedman test.

^c^WMFT: Wolf Motor Function Test.

^d^FMA-UE: Fugl-Meyer Assessment of Upper Extremity.

^e^BDI: Beck Depression Inventory.

^f^ROM: range of motion.

## Discussion

### Principal Findings

In this study, we performed a comprehensive assessment based on a ML algorithm and wearable device. We developed an HBR system using a commercial smartwatch with the ML model and evaluated the effectiveness of the HBR system via a clinical trial. The ML model based on a CNN algorithm showed good to excellent accuracy ranging from 86.5% to 100%, and the clinical trial showed a relevant increase in ROM and the WMFT function score.

While developing the HBR system using a commercial smartwatch, determining the types of sensors that provide maximal accuracy was an important issue. According to previous research that used an IMU sensor for activity recognition, an accelerometer is the most accurate sensor for activity recognition [29,30,33-36]. Based on the results of the cross-validation test in our study, the accelerometer signal combined with gyroscope findings provided the most accurate results. This is consistent with the result in the study by Hyunh et al [37], which attempted to detect falls by using an IMU sensor at the chest. It was reported that adding a gyroscope can reduce the false-positive rate and increase specificity from 82.72% to 96.20%. However, in our study, the difference in the accuracy of the results obtained when using an accelerometer and an accelerometer combined with a gyroscope was relatively small (1.1%-1.8%), and in the case of active scapular exercises, the accuracy of the gyroscope was even higher than that of other approaches. Thus, we believe that the choice of the most accurate sensor may depend on the type of exercise and the location of the sensor. Our research, which involved the detection of repetitive and slow home exercise tasks by a smartwatch, showed that the combination of an accelerometer and a gyroscope provided the most accurate signal. However, considering that the improvement in accuracy with the addition of a gyroscope was relatively small and that the addition required doubled computation and battery loading for the gyroscope, we believe that an accelerometer-only signal could be an alternative choice.

Since the learning data set is a decisive factor in optimizing the ML algorithm, we compared the accuracy of the ML model built with personal data and that built with total data. The ML model based on total data was built with data from all participants and the ML model based on personal data was implemented by using the participants’ own data. Although the amount of total data was larger than that of personal data, each exercise motion in the total data set represents a mix of different motions of all participants. Therefore, through this comparison, we attempted to determine whether the quantity or quality of data is important. According to the results, we found that quality was more important. The ML model built with only personal data (99.9%), which represented the quality of data, was more accurate than the ML model built with total data (95.4%), which represented the quantity of data. This means that data personalization was more important than the total amount of data, especially for chronic stroke patients who had various disabilities and individual motion characteristics. We think that the different exercise motions of other patients contaminated the data consistency and had a bad influence on the ML model [38].

With regard to the clinical trial, the HBR group showed significant functional recovery (mean difference=2.8, *P*=.02) in the WMFT. However, FMA-UE did not show significant results (mean difference=1.9, *P*=.46). We think this is related to the different traits of both functional assessment methods. FMA-UE (total 66 scores) is an assessment tool for identifying motor impairment, and it involves reflex activity (6), flexor synergy (12), extensor synergy (6), combining synergy (6), movement out of synergy (6), wrist (10), hand (14), and coordination/speed (6) on an ordinal scale from 0 to 2 (0, none; 1, partial; 2, complete). In contrast, the WMFT (total 75 scores) is a test for assessing functional performance, providing insight into joint-specific and integrative limb movements graded from 0 to 5, with 15 function-based tasks [39]. According to the study performed by Wolf et al [40], the WMFT is more sensitive than FMA-UE for assessing functional improvement in less affected stroke patients. Thus, the different results of the two functional tests indicate that the home rehabilitation exercise for 12 weeks had beneficial effects in functional recovery, but it was not enough to change synergic movement or hand and wrist function.

In terms of shoulder ROM, we found a significant increase in shoulder flexion (*P*=.02) and internal rotation ROM (*P*=.001) in the HBR group by the Friedman test. According to the post-hoc analysis involving the Wilcoxon signed-rank test with Bonferroni correction, significant increases in the first 6 weeks of home exercise were noted for shoulder joint ROM with flexion (*P*=.004) and internal rotation (*P*=.001). However, there was no change in external rotation and extension ROM. Regarding the reasons for ROM increase, we think it is associated with the exercise protocol of our study. Among the four kinds of home exercises in our study protocol, bilateral flexion, wall push, and towel slide required wide movements of shoulder flexion and internal rotation. The exercise time records from our HBR system support this since patients performed the shoulder bilateral flexion exercise for the longest time when compared with other exercises. The shoulder extension ROM exercise was not included in our home exercise protocol. Although shoulder external rotation is required to perform the active scapular exercise, the external rotation ROM was not increased. This result is associated with the fact that chronic stroke patients usually have internally rotated joint contractures associated with impaired motor synergy [41,42]. We consider that the absence of a relevant change in the hand grip test was also related with the fact that our home exercise protocol required wide movement of the shoulder joint, but less motion of the wrist and hand joints.

The benefit of the HBR system was not only clinical improvement but also a decreased drop-out rate, which might encourage the application of wearable systems for HBR. We found that the drop-out rate was lower in the HBR group than in the control group at 12 weeks (5/22, 22% vs 4/10, 40%) and 18 weeks (10/22, 45% vs 10/10, 100%). After this study was completed, we interviewed two participants who dropped out to find out why they decided not to continue the study. They said that they became less interested in the conventional home rehabilitation program because the weekly phone calls did not help bring about any visible improvement and bothered them. We think the HBR system has a good influence on the motivation for home exercise and the relationship with physical therapists. According to the self-determination theory, which refers to each person’s ability to make choices and manage their own life [24], people need to experience a sense of belonging and attachment with others, which is called “connection or relatedness.” In addition, people need to feel in control of their own behaviors and goals, which is called “autonomy.” Our HBR system would assist patients to record an exercise time (autonomy) and to communicate with a clinician (connection or relatedness).

Regarding the depression index, previous randomized controlled trials reported that home rehabilitation can reduce the incidence of depression [43,44]. However, BDI in our study did not show a relevant difference. It only showed a trend of positive effects (*P*=.06). The finding might be significant with a greater number of participants or a longer period because our protocol was relatively shorter than the period in the literature [43,44].

Lastly, there was no relevant difference in the HBR group between 12 weeks and 18 weeks (6 weeks after the final assessment without the HBR system). However, we believe that the HBR system is more effective when used consistently in home care because most of the clinical outcomes at 18 weeks showed a decreasing trend compared with 12 weeks.

### Comparison With Prior Work

Previously, Wade emphasized that enabling self-directed practice is critical for stroke rehabilitation [45]. Regarding the strategy of self-directed practice, it has been shown that verbal encouragement does not have an impact on increasing rehabilitation activity after stroke [46]. Therefore, various methods of self-management training for upper limb rehabilitation have been suggested, including robot-assisted therapy. For example, Markopoulos et al [47] and Holden et al [48] developed a watch-like device. They used a visual feedback system as a self-management tool, but there was no remote supervision with the therapist. The mobile health system proposed by Dobkin [17] uses a similar strategy as that of our approach, which is called the rehabilitation internet-of-things (RIoT) device. However, we applied the ML model for home exercise detection and used a commercial smartwatch to simplify the user device interface, which has been regarded by previous researchers as the most important factor for use in clinical practice [17,49].

With regard to robot-assisted therapy, Lo et al [50] reported that robot-assisted therapy showed no relevant difference at 12 weeks and only showed improvement over 36 weeks when compared with typical care. Additionally, it costed US $15,562 for the 36-week program [50]. In contrast, our HBR system increased flexion ROM at 6 weeks and showed improvement of the WMFT score at 12 weeks. Considering the treatment cost of robot-assisted therapy, our HBR system strategy could be a better treatment modality with similar clinical improvement.

### Limitations

There are several limitations in this study. First, the total number of patients who completed our program was relatively small to derive statistically strong evidence, particularly in the control group. Further work with a larger sample size would be helpful for more confirmative conclusions. Second, there was a discrepancy in the number of participants in the control and HBR groups. Only six participants from the control group were enrolled in the data analysis process. However, while carrying out our research, the loss of participants was inevitable in the control group because they were tired of receiving calls regarding management without any benefit, indicating the limitation of a conventional method. Third, there could have been loss of time measurement in the HBR group since some patients stated that they sometimes performed home exercise without the smartwatch owing to the inconvenience of wearing the smartwatch. Therefore, we think the exercise time recorded in the database was an underestimation of the real home exercise time. Fourth, the actual accuracy of exercise detection at home was not assessed. Although some researchers have attempted to address the privacy preservation of sensitive personal data based on a deep learning algorithm [51], we did not implement this approach and only calculated the accuracy based on a five-fold cross-validation test. Therefore, the actual accuracy, which is the correct prediction rate of exercise detection at home, could not be assessed because all patients wanted to protect their privacy. Fifth, there could have been selection bias associated with the positions of local health centers at different locations. Although we cannot quantify the difference, we think the bias was not relevant enough because both centers are closely located (50 km away) and the socioeconomic status is similar.

### Conclusions

This study found that a home care system using a commercial smartwatch and ML model can facilitate participation in home training and improve the functional score of the WMFT and shoulder ROM of flexion and internal rotation in the treatment of patients with chronic stroke. We recommend our HBR system strategy as an innovative and cost-effective home care treatment modality.

## Appendix

Multimedia Appendix 1 Home-based rehabilitation system application video.

Multimedia Appendix 2 App view of home exercise results for the user and supervisor.

Multimedia Appendix 3 Accuracy calculation by the cross-validation test.

Multimedia Appendix 4 Clinical results with the home-based rehabilitation system.

Multimedia Appendix 5 Comparison between 12 weeks and 18 weeks in the home-based rehabilitation group.

Multimedia Appendix 6 CONSORT-eHEALTH checklist (V 1.6.1).

## Abbreviations

BDI: Beck Depression Inventory
CNN: convolutional neural network
FMA-UE: Fugl-Meyer Assessment of Upper Extremity
HBR: home-based rehabilitation
IMU: inertial measurement unit
ML: machine learning
ROM: range of motion
WMFT: Wolf Motor Function Test
