# Supplementary material for: Development and Clinical Evaluation of a Web-Based Upper Limb Home Rehabilitation System Using a Smartwatch and Machine Learning Model for Chronic Stroke Survivors: Prospective Comparative Study
Source: JMIR Mhealth Uhealth. 2020 Jul 9;8(7):e17216. doi: 10.2196/17216 (PMC7380903; doi:10.2196/17216)
Supplement: Multimedia Appendix 2 [file mhealth_v8i7e17216_app2.pdf]

Multimedia Appendix 2. Application view of home exercise results for user and supervisor

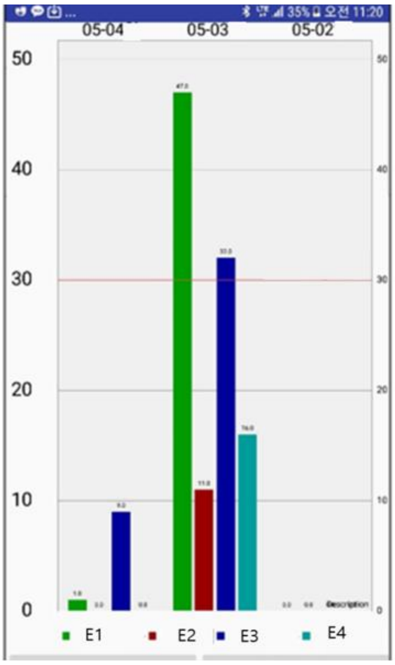

Screenshot of user app.

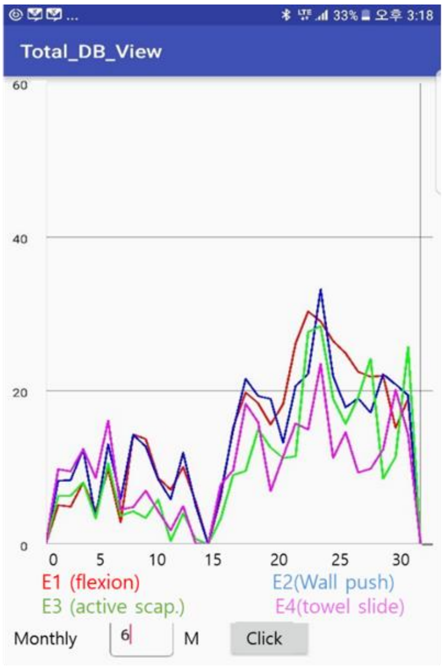

Screenshot of therapist app.
