# Supplementary material for: Development and Clinical Evaluation of a Web-Based Upper Limb Home Rehabilitation System Using a Smartwatch and Machine Learning Model for Chronic Stroke Survivors: Prospective Comparative Study
Source: JMIR Mhealth Uhealth. 2020 Jul 9;8(7):e17216. doi: 10.2196/17216 (PMC7380903; doi:10.2196/17216)
Supplement: Multimedia Appendix 3 [file mhealth_v8i7e17216_app3.pdf]

Multimedia Appendix 3. Accuracy calculation by cross-validation test

Table 1-1. Personal data (A)

	TP	FN	FP	TN	Accuracy
No exercise	399	0	0	825	100 (1224/1224)[a]
Bilateral flexion	294	5	12	809	98.5 (1103/1120)
Wall push	255	3	7	759	99.0 (1014/1024)
Active scapula	274	35	43	756	93.0 (1030/1108)
Towel slide	293	0	0	832	100 (1125/1125)
Total	1515	43	62	3981	98.1 (5496/5601)

Appendix Table 1-2. Personal data (G)

	TP	FN	FP	TN	Accuracy
No exercise	378	21	31	794	95.8 (1172/1224)
Bilateral flexion	280	19	6	815	97.8 (1095/1120)
Wall push	221	37	28	738	93.7 (959/1024)
Active scapula	261	11	16	820	97.6 (1081/1108)
Towel slide	272	21	30	802	95.5 (1074/1125)
Total	1412	109	111	3969	96.0 (5381/5601)

Appendix Table 1-3. Personal data (A+G)

	TP	TN	FP	TN	Accuracy
No exercise	399	0	0	825	100 (1224/1224)
Bilateral flexion	290	9	2	819	99.0 (1109/1120)
Wall push	258	0	0	766	100 (1024/1024)
Active scapula	272	0	0	836	100 (1108/1108)

Towel slide	293	0	0	832	100 (1125/1125)
Total	1512	9	2	4078	99.9 (5590/5601)

Appendix Table 2-1. Total data (A)

	TP	TN	FP	TN	Accuracy
No exercise	388	11	21	804	97.4 (1192/1224)
Bilateral flexion	274	25	19	802	96.1 (1076/1120)
Wall push	224	34	30	736	93.8 (960/1024)
Active scapula	228	44	42	794	92.2 (1022/1108)
Towel slide	258	35	30	802	94.2 (1060/1125)
Total	1372	149	142	3938	94.8 (5310/5601)

Appendix Table 2-2. Total data (G)

	TP	TN	FP	TN	Accuracy
No exercise	389	10	17	808	97.8 (1197/1224)
Bilateral flexion	287	12	9	812	98.1 (1099/1120)
Wall push	200	58	80	686	86.5 (886/1024)
Active scapula	198	74	70	766	87.0 (964/1108)
Towel slide	228	65	62	770	88.7 (998/1125)
Total	1302	219	238	3842	91.8 (5144/5601)

Appendix Table 2-3. Total data (A+G)

	TP	TN	FP	TN	Accuracy
No exercise	399	0	0	825	100 (1224/1224)
Bilateral flexion	282	17	14	807	97.2 (1089/1120)
Wall push	226	32	38	728	93.2 (954/1024)

Active scapula	233	39	39	797	93.0 (1030/1108)
Towel slide	268	25	26	806	95.5 (1074/1125)
Total	1408	113	117	3963	95.8 (5371/5601)
