# Supplementary material for: Development and Clinical Evaluation of a Web-Based Upper Limb Home Rehabilitation System Using a Smartwatch and Machine Learning Model for Chronic Stroke Survivors: Prospective Comparative Study
Source: JMIR Mhealth Uhealth. 2020 Jul 9;8(7):e17216. doi: 10.2196/17216 (PMC7380903; doi:10.2196/17216)
Supplement: Multimedia Appendix 5 [file mhealth_v8i7e17216_app5.pdf]

Multimedia Appendix 5. Comparison between 12 weeks and 18 weeks in HBR Group.

	HBR group (N = 17)	HBR group (N = 12)	
Evaluation time (weeks)	12 weeks[e] (Final assessment) Mean(SD)	18 weeks (6 weeks after final assessment) Mean(SD)	*P*-value[f]
**Functional assessment test**			
WMFT[a]	42.5(23.7))	35.1(25.0)	.08
FMA-UE[b]	38.5(18.3)	38.5(19.0)	.51
Grip power (Kg)	14.8(12.1)	11.4(9.7)	.33
BDI[c]	8.0(9.9)	8.9(9.5)	.72
**Shoulder ROM[d]**			
Flexion	94.7(48.9)	88.4(50.1)	.35
Extension	34.7(19.9)	34.6(19.5)	.68
Internal rotation	63.5(26.9)	62.5(33.6)	.50
External rotation	16.9(18.4)	12.5(15.7)	.12

[a]Wolf Motor Function Test; [b]Fugl-Meyer Assessment for upper extremity; [c]Beck Depression Inventory;[d]Range of Motion; [e]After 12 weeks, the HBR system was retrieved from patients [f]Statistically significant compared by Wilcoxon signed rank test.
